# Peri-Procedural Management of Direct-Acting Oral Anticoagulants (DOACs) in Transcatheter Miniaturized Leadless Pacemaker Implantation

**DOI:** 10.3390/jcm12144814

**Published:** 2023-07-21

**Authors:** François Diederik Regoli, Ardan M. Saguner, Angelo Auricchio, Andrea Demarchi, Elena Pasotti, Giulio Conte, Maria Luce Caputo, Tardu Özkartal, Alexander Breitenstein

**Affiliations:** 1 Service of Cardiology, Hospital of San Giovanni, Cardiocentro Ticino Institute, 6500 Bellinzona, Switzerland; 2 Cardiology Department, Cardiocentro Ticino Institute, 6900 Lugano, Switzerland; 3 University Heart Center Zurich, University Hospital Zurich, 8091 Zurich, Switzerland

**Keywords:** direct-acting oral anticoagulants’ management, leadless pacing, anticoagulation management, leadless pacemaker implantation, bleeding complications in leadless pacemaker

## Abstract

Introduction: Data on peri-operative management of direct-acting oral anticoagulants (DOACs) during transcatheter pacing leadless system (TPS) implantations remain limited. This study aimed to evaluate a standardized DOAC management regime consisting of interruption of a single dose prior to implantation and reinitiation within 6–24 h; also, patient clinical characteristics associated with this approach were identified. Method: Consecutive patients undergoing standard TPS implantation procedures from two Swiss tertiary centers were included. DOAC peri-operative management included the standardized approach (Group 1A) or other approaches (Group 1B). Results: Three hundred and ninety-two pts (mean age 81.4 ± 7.3 years, 66.3% male, left ventricular ejection fraction 55.5 ± 9.6%) underwent TPS implantation. Two hundred and eighty-two pts (71.9%) were under anticoagulation therapy; 192 pts were treated with DOAC; 90 pts were under vitamin-K antagonist. Patients treated with DOAC less often had structural heart disease, diabetes mellitus, and advanced renal failure. The rate of major peri-procedural complications did not differ between groups 1A (*n* = 115) and 1B (*n* = 77) (2.6% and 3.8%, *p* = 0.685). Compared to 1B, 1A patients were implanted with TPS for slow ventricular rate atrial fibrillation (AF) (*p* = 0.002), in a better overall clinical status, and implanted electively (<0.001). Conclusions: Standardized peri-procedural DOAC management was more often implemented for elective TPS procedures and did not seem to increase bleeding or thromboembolic adverse events.

## 1. Introduction

The use of leadless pacemaker technology has remarkably increased over the last few years [1]. The most commonly used technology is the Micra transcatheter pacemaker system (TPS; Medtronic, MN, USA), a small capsule-shaped device that is implanted into the wall of the right ventricle from through the femoral vein via a 27 F transvenous sheath. Presently, TPS functions as a single-chamber device in either VVI or VDD modalities. Registry data have repeatedly shown that this system reduces device-related peri-procedural and long-term morbidity compared to conventional transvenous lead pacemakers [2,3,4,5]. According to data from the Micra IDE study, as well as the post-approved Micra registry, the incidence of major peri-procedural bleeding events ranges between 1.4 and 3% [2,6]. Importantly, a recent contribution by Piccini and colleagues [5] reported that pericardial effusion during TPS positioning was associated with the following characteristics: body mass index ≤ 20 kg/m^2^, age > 85 years, female gender, chronic obstructive pulmonary disease, and a history of atrial fibrillation (AF). Given the concern for major bleeding complications, especially in high-risk patients, adequate peri-procedural anticoagulation management during TPS positioning is of utmost relevance. 

In a single-center, retrospective study, Kiani and colleagues [7] reported TPS implantation under uninterrupted vitamin-K antagonist (VKA). In this limited series (26 patients), no increased bleeding events were found with this strategy. In another retrospective study [8], including a higher number of patients under DOAC therapy, in most patients (89%), the DOAC was stopped prior to the procedure. In five patients, the implantation procedure was performed without interrupting the DOAC. The safety and feasibility of an uninterrupted anticoagulation peri-procedural approach have been further supported by a large multicenter cohort that included 1210 patients under chronic anticoagulation [9]. This study showed no difference between interrupted or uninterrupted ACO management strategies. However, in this large study, the specific type of anticoagulation therapy, whether VKA or DOAC, was not specified.

Considering specific peri-procedural management of DOAC, European Heart Rhythm Association (EHRA) recommendations [10,11] advise that in patients undergoing an elective invasive low bleeding risk intervention, such as TPS positioning, DOAC intake should be stopped ≥24 h prior to the procedure and reinitiated 6–24 h after the procedure. Whether this DOAC peri-procedural management scheme is also adequate for TPS implantation, a procedure involving femoral transvenous catheterization with a large-sized 27 F venous sheath, remains an open question. 

This retrospective two-center study investigates peri-procedural DOAC management during leadless pacemaker implantation. The study objectives are two-fold:

1. To assess the effects of a *standardized* DOAC management approach, consisting in withholding one dose prior to the procedure and reinitiation 6–24 h, on peri-procedural adverse events;
2. To identify clinical characteristics associated with this DOAC management strategy.

## 2. Materials and Methods

This two-center retrospective study included all consecutive patients undergoing TPS implantation at Cardiocentro Ticino (Lugano) and at the University Hospital of Zurich between September 2015 and December 2021. Study data were included in an institutional pacemaker database, approved by the regional ethics review board (KEK-ZH-NR: 2020-00811). Baseline demographic and clinical characteristics were assessed, including indications for single-chamber pacemakers, major comorbidities, and ongoing treatments. For each patient taking oral anticoagulants, the peri-procedural management of anticoagulation was assessed (see section below). 

### 2.1. Definition of Peri-Procedural Anticoagulation Regimens and General Patient Management 

Different anticoagulation therapy regimens were prescribed based on patient bleeding and thromboembolic risk profiles, institutional protocol, and operator discretion. 

Concerning DOAC (Group 1), two subgroups of peri-procedural management were considered:

- Group 1A included patients treated with the *standardized* approach, which consisted of DOAC interruption of 1 dose prior to the procedure, irrespective of the half-life of the drug. The therapy was then reinitiated 6–24 h after the procedure;
- Group 1B gathered all other peri-procedural DOAC regimens, including:
  - *Interrupted and delayed reinitiation:* DOAC interruption before the procedure of at least 2 consecutive doses for either dabigatran/apixaban or for rivaroxaban/edoxaban. DOAC anticoagulation therapy was then reinitiated >24 h after the procedure;
  - *Uninterrupted*: no peri-procedural DOAC interruption was performed;
  - *Interrupted with “Bridging”*: in patients treated with DOACs, the oral anticoagulant was interrupted before the procedure for at least 2 consecutive doses when treated with dabigatran/apixaban or 1 dose for rivaroxaban/edoxaban, and either fractionated or unfractionated heparin was administered peri-procedurally.

For patients who were under chronic VKA (Group 2), ACO was managed as follows:

- *Interrupted with “Bridging”*: in patients under chronic VKA, this approach consisted in suspending VKA at least 72 h before, performing TPS positioning with INR < 1.5, and either fractionated or unfractionated heparin was administered peri-procedurally;
- *Interrupted without “Bridging”*: in patients under chronic VKA, this approach consisted in suspending VKA at least 72 h before, performing TPS positioning with INR < 1.5, and reinitiating VKA after the procedure without resorting to “bridging” with either fractionated or unfractionated heparin;
- *Uninterrupted*: TPS positioning was performed without discontinuing VKA with INR level < 3 [12].

After TPS positioning, bed rest was prescribed for at least 8 h, including 6 h with a groin pressure dressing. 

### 2.2. Leadless Pacemaker Implantation 

Implantations were performed with local anesthesia and mild sedation in line with previous reports [13,14]. The procedure was performed according to a standardized implantation protocol, which has been presented in detail elsewhere [13]. Briefly, through femoral vein access and following appropriate dilation at the level of the right groin, the 27 Fr venous sheath was advanced into the right atrium over a super-stiff guidewire. The steerable delivery system was gently advanced to the right atrium and curved across the atrioventricular junction to reach the right ventricular septum. Contrast dye injection verified adequate contact to the inferior or mid-septal endocardial border of the right ventricle before delivery of the device. After verifying device fixation and confirming adequate electrical measures, the tether was cut, and the delivery system was removed. Hemostasis at the femoral puncture site was obtained either by applying a figure-of-eight suture or with the use of vascular closure devices (Perclose ProGlide™ Suture-Mediated Closure System, Abbott, Chicago, IL, USA) [15]. The choice of the hemostasis technique was left to the operator’s discretion. 

### 2.3. Study Endpoints and Classification of Adverse Events

Peri-procedural adverse events were considered as those events occurring within 30 days of the procedure. Major complications included any intra-procedural and peri-procedural adverse events causing transitory or permanent functional impairment that required additional unplanned measures for resolution. Minor complications included any mild adverse event that caused transient patient discomfort, causing a prolongation of hospital stay, but that did not require additional medical measures for resolution. 

### 2.4. Statistics

Data are described as mean and standard deviation or median and 25th–75th percentiles if continuous (depending on distribution), and counts and percent if categorical. For comparison between groups, the Student’s *t*-test was performed for normally distributed continuous variables and the Mann–Whitney-U test for non-parametric data. Fisher´s exact test was used to compare group distributions of categorical variables. A 2-sided *p*-value < 0.05 was considered statistically significant. All analyses were performed using Stata, version 15 (StataCorp, College Station, TX, USA).

## 3. Results 

### 3.1. Patient Characteristics and Management of Anticoagulation during TPS

**Figure 1:**
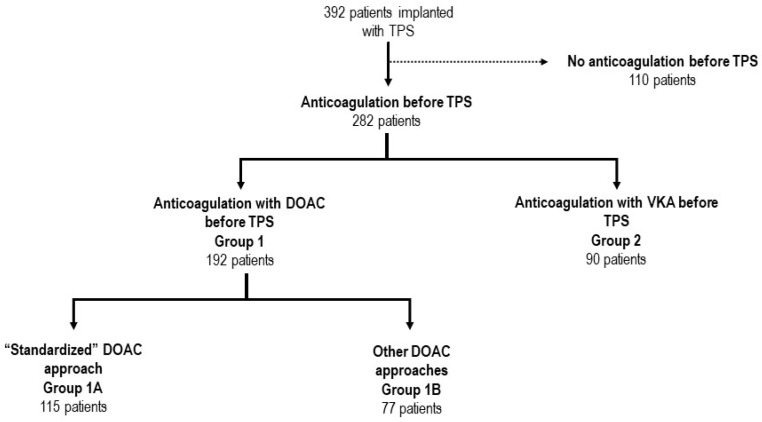
Study flow diagram presenting the different groups based on the type of anticoagulation therapy prescribed. Patients under chronic anticoagulation therapy with a DOAC (Group 1) were divided according to the standardized approach (Group 1A) or other DOAC management strategies (Group 1B).

**Figure 2:**
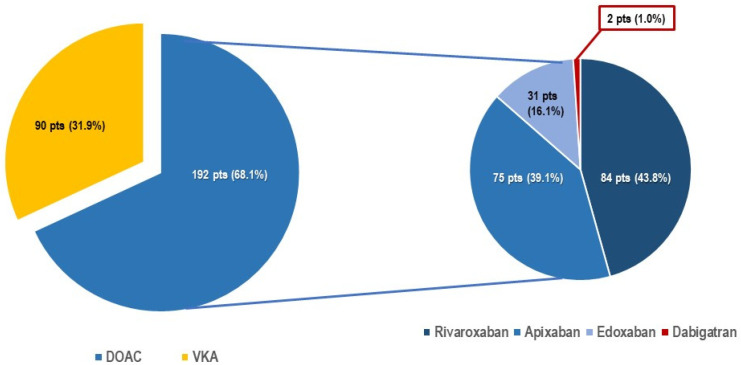
On the left, the pie graph presents the distribution of patients under chronic anticoagulation therapy divided into either DOAC or VKA. The pie graph on the right highlights the specific distribution of DOAC drug agents.

**Figure 3:**
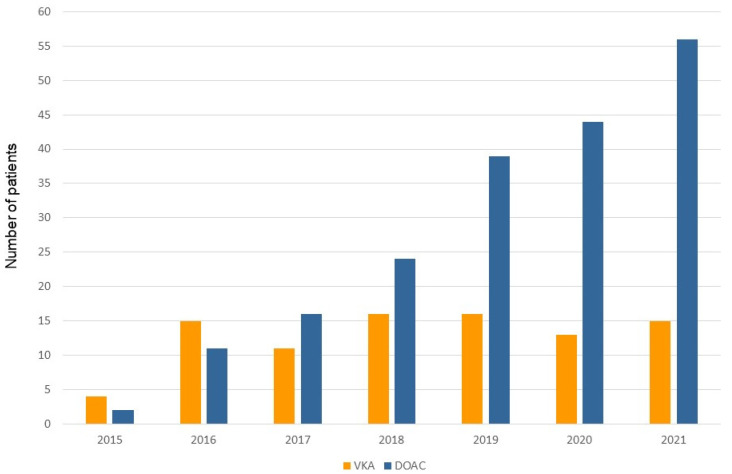
Distribution of patients treated with TPS from 2015 to 2021 under chronic anticoagulation therapy with either a DOAC (blue columns) or VKA (yellow columns).

Three-hundred and ninety-two consecutive patients undergoing leadless pacemaker implantation were included in this analysis. The mean age was 81.4 ± 7.3 years, and the majority of patients were male (66.3%) (Table 1). Structural heart disease was present in 71.2% of patients, and the mean left ventricular ejection fraction (55.5 ± 9.6%) was preserved. Most patients suffered from one or more associated comorbidity, including renal impairment (53.1%), diabetes mellitus (19.6%), chronic obstructive lung disease (15.3%), tumoral disease (14.5%), peripheral artery disease (14.5%), and/or high bleeding risk (7.2%). The main indication for device implantation was bradycardic atrial fibrillation (40.3%). Of note, 282 patients (71.9%) were under oral anticoagulation, including 192 (68.1%) patients (Group 1) treated with DOAC (Figure 1 and Figure 2) and 90 patients (31.9%) under vitamin K antagonists (VKA) (Group 2). Peri-procedural management strategies of VKA oral anticoagulation included interruption 3–5 days prior to the procedure and “bridging” with heparin (47 patients, 52.2%), interruption without “bridging” (25 patients, 27.7%), or performing TPS implantation under uninterrupted VKA (*n* = 18, 20%). The use of DOAC has gradually and continuously increased over time (Figure 3). Patients under DOAC less often presented underlying structural heart disease (DOAC: 55.4% vs. VKA: 95.3%, *p* < 0.001) and associated comorbidities, including diabetes mellitus (DOAC: 16.0% vs. VKA: 21.9%, *p* = 0.001), advanced renal failure requiring hemodialysis (DOAC: 0 vs. VKA: 7.8%, *p* < 0.001), and higher bleeding risk requiring left appendage closure (DOAC: 1.1% vs. VKA 10.9%, *p* < 0.001). Major (DOAC: 3.1% vs. VKA: 2.2%, *p* = 1.000) and minor (DOAC: 1.6% vs. VKA: 3.3%, *p* = 0.388) complications at 30 days did not differ between the two groups. 

### 3.2. Differing Peri-Procedural DOAC Regimens during TPS Procedure

Most patients under DOAC anticoagulation were treated according to the standardized approach (*n* = 115 patients, 59.9%, Group 1A) (Figure 1). There were some differences in the baseline characteristics between the two groups (Table 2). Patients in group 1A presented significantly less structural heart disease (1A: 42.6% vs. 1B: 68.8%, *p* < 0.001), fewer comorbidities like renal insufficiency (1A: 33.0% vs. 1B: 66.2%, *p* < 0.001) and/or peripheral artery disease (1A: 4.3% vs. 1B: 20.8%, *p* < 0.001). Furthermore, there were some differences in pacemaker indication between the groups, implantation in Group 1A being more often performed to treat slow-ventricular rate atrial fibrillation (1A: 55.7% vs. 1B: 32.5%, *p* = 0.002) and the DOAC most often prescribed in Group 1A patients was rivaroxaban (1A: 54.7% vs. 1B: 27.2%, *p* < 0.001), while apixaban was more often prescribed in group 1B patients (apixaban use: 1A 31.3% vs. 1B: 50.6%, *p* = 0.010). For other differences in pacemaker indication, refer to Table 2. Noteworthy, the implantation procedure was performed more often in the setting of a planned hospitalization for Group 1A patients compared to Group 1B (1A: 57.4% vs. 1B: 23.4%, *p* < 0.001). 

In Group 1A, the DOAC was stopped with a mean of 25.7 ± 8.5 h before and reinitiated at 7.4 ± 5.9 h after the procedure (Table 3). In patients from group 1B, peri-procedural management of DOAC varied considerably, and included “*interruption and delayed reinitiation*” (*n* = 54, 70.1%), “*interruption and bridging with heparin*” (*n* = 13, 16.9%), and “*uninterrupted*” (*n* = 10, 13.0%). Less delay was observed in Group 1A for both peri-procedural interruption and reinitiation. The mean interruption was 21.4 ± 5.2 h before the procedure for Group 1A and 27.0 ± 27.1 h for Group 1B (*p* = 0.032); the mean reinitiation delay after the procedure was 7.4 ± 5.9 for Group 1A compared to 27.7 ± 16.8 h for Group 1B (*p* < 0.001). 

### 3.3. Differing Peri-Procedural DOAC Regimens and Clinical Outcomes

Procedural and pre-discharge characteristics are presented in Table 3. No differences were found in terms of procedural efficacy, including successful leadless pacemaker implantation (100 % in both groups; *p* = 1.000). Major intra- and post-procedural complications up to 30 days after discharge were low and did not differ between the two groups (1A 2.6% vs. 1B 3.8%, *p* = 0.685). The occurrence of minor groin site hematoma was also similar between the two groups (1A: 5.2% vs. 1B: 3.9%, *p* = 0.743). Median length of hospital stay was significantly lower in Group 1A compared to 1B (1A: 3.0 [IQR 2.0–4.8] vs. 1B: 4.0 [IQR 3.0–12.5] days, *p* = 0.019).

## 4. Discussion

In this retrospective study, patients undergoing leadless pacemaker implantation in two Swiss tertiary institutions were included. Almost one in every two patients in this cohort was under chronic oral anticoagulation therapy with DOAC. A *standardized* peri-procedural DOAC therapy regimen (interruption of one dose prior to the intervention, irrespective of the half-life of the drug, with reinitiation 6–24 h after the procedure) had comparable peri-procedural clinical outcomes compared to other different peri-procedural DOAC management approaches.

### 4.1. The Growing Importance of DOAC during TPS Procedure

In the experience reported herein, which extends from September 2015 to the end of 2021, more than two-thirds of anticoagulated patients (68.1%) undergoing device implantation were under DOAC. This number is considerably higher compared with previously reported other single-center cohorts [7,8]. San Antonio and colleagues [8] reported that only around 20% of anticoagulated patients were under DOAC. One possible explanation for this difference is the timeline of patient inclusion. The two above-cited series [7,8] included patients between the years 2014 and 2018 compared to 2015 and 2021 in our cohort. There is a clear shift from VKA to DOAC over time which is even more predominant in the last two years (Figure 3). This trend can be explained by the growing amount of evidence outlining the safety and efficacy of DOAC in high-risk patient subgroups [16], including patients with mild-to-moderate renal impairment [11,17,18], as well as patients with stable coronary artery disease [8,16]. In fact, no difference was found in the proportion of patients presenting renal impairment or structural heart disease between patients treated with DOAC and those treated with VKA. Furthermore, the preferential use of DOAC in patients suffering from atrial fibrillation is also underscored in the most recent European guidelines on the management of atrial fibrillation [16].

### 4.2. Peri-Procedural Management of DOAC during TPS Procedure: Which Is the Best Approach?

Peri-procedural management of anticoagulation is a relevant issue in patients undergoing leadless pacemaker implantation, especially in regard to the number of frail patients in need of such an intervention. According to the EHRA practical recommendations for DOAC management [10,11], leadless pacemaker implantation could be considered an intervention with low bleeding risk, since clinically important bleeding is usually infrequent and controllable. As such, the recommendations propose skipping one DOAC dose prior to the procedure and reinitiation on the same day (generally between 6 and 8 h after the procedure). Although it may be difficult to extend these recommendations to leadless pacemaker implantation given the limited outcome data available, there is, however, substantial evidence for a shorter anticoagulation interruption timing in patients undergoing conventional transvenous device implantation and box change [10,11]. Indeed, both Bruise CONTROL trials I–II [12,19] demonstrated that careful intraoperative hemostasis of the device pocket allows effective control of bleeding in patients with either uninterrupted VKA or DOAC. However, during implantation of standard CIED transvenous systems, the main source of bleeding is the subclavicular subcutaneous pouch. Given the fact that the leadless pacemaker implantation procedure implicates a completely different implantation technique compared to transvenous pacemakers, the extension of the results of the Bruise CONTROL 2 trial [19] for the peri-procedural management of DOAC during TPS is rather inappropriate. 

San Antonio and colleagues [8] followed a similar management protocol as the one reported herein for the standardized approach consisting in discontinuing DOAC 12–24 h before the procedure and reinitiating the medication 6–24 h after. In the series reported by Kiani et al. [7], two different peri-procedural DOAC management strategies were pursued: The more commonly practiced approach (*n* = 36 patients) consisted in interrupting dabigatran or apixaban for at least two consecutive doses (rivaroxaban was withheld for at least one dose) before the procedure. The second approach, performed in only five patients, consisted of withholding apixaban/dabigatran for one or fewer consecutive pre-procedural doses or no dose interruption for rivaroxaban. The incidences of major and minor complications in this series were overall low, indicating appropriate anticoagulation management; however, the low number of patients limits the clinical impact of these data. 

In our experience, for the patient cohort treated with a standardized peri-procedural approach, three major complications occurred and did not differ compared to other approaches. The standardized approach was most often the approach of choice for peri-procedural management of DOAC in patients with a planned TPS procedure, presenting better overall clinical status in whom the intervention could be performed on the same day or the day after admission. Administration of DOAC within 6–24 h after the procedure and early mobilization may control thromboembolic risk. Several studies have reported that early mobilization after cardiac catheterization is safe and may potentially reduce hospitalization duration [20,21]. Conversely, patients treated with other peri-procedural DOAC regimens (Group 1B) were usually patients with prolonged hospitalization, limited mobility, overall poorer clinical status, and who required a pacemaker urgently. In these patients, DOAC management was heterogenous, based on patient-specific risk profiles and procedural urgency. 

### 4.3. Study Limitations

This is a retrospective two-center study, gathering patients chronically treated with DOAC therapy undergoing implantation of a leadless pacing system. The lack of a clearly defined control group to compare the standardized approach represents a limitation. Moreover, the patient cohort remains small, and the rate of major complications is low. The scope of the present study was to provide some preliminary, hypothesis-generating findings for the optimization of peri-procedural DOAC management during TPS, given the limited data in this field. 

## 5. Conclusions

Standardized peri-procedural management of DOAC during TPS implantation consisting in skipping a single DOAC dose before the procedure and reinitiating the therapy within 6–24 h, does not increase bleeding and thromboembolic events. This approach was prescribed more frequently in patients presenting a good overall clinical status, who were not pacemaker-dependent, and who underwent an elective procedure. Prospectively designed studies would be useful to further optimize the management of DOAC during TPS. 

## Figures and Tables

**Table 1:** Baseline characteristics of patients implanted with the transcatheter single-chamber pacemaker system.

Characteristics	All (*n* = 392)	DOAC (Group 1, *n* = 192)	VKA (Group 2, *n* = 90)	*p* Value
**Demographic, clinical**				
**Age, years**	81.4 ± 7.3	81.2 ± 7.2	81.7 ± 7.4	0.928
**Male**	260 (66.3)	118 (60.9)	63 (70.0)	0.184
**Structural heart disease**	279 (71.2)	112 (58.3)	88 (97.7)	<0.001
**Ischemic**	145 (37.0)	58 (30.2)	39 (43.3)	
**Valvular**	111 (28.3)	41 (21.3)	43 (47.8)	
**Other**	23 (6.0)	13 (6.8)	6 (6.7)	
**Hypertension**	334 (85.2)	173 (90.2)	84 (93.6)	0.501
**Diabetes mellitus**	77 (19.6)	31 (16.0)	31 (21.9)	0.001
**Renal impairment (≥1.5 mg/dL)**	208 (53.1)	82 (42.9)	49 (54.6)	0.074
**Dialysis**	34 (8.7)	0	7 (7.8)	<0.001
**Chronic obstructive lung disease**	60 (15.3)	29 (15.1)	9 (10.0)	0.268
**Peripheral artery disease**	57 (14.5)	21 (10.7)	10 (11.1)	1.000
**Previous stroke**	53 (13.5)	25 (13.2)	13 (14.4)	1.000
**Tumoral disease**	60 (15.3)	29 (15.2)	13 (14.1)	1.000
**Other comorbidities**	8 (2.0)	6 (3.1)	1 (1.1)	0.437
**Left ventricular ejection fraction (%)**	55.5 ± 9.6	55.7 ± 8.9	55.1 ± 9.7	0.609
**Planned hospitalization for implantation**	156 (39.8)	83 (43.5)	30 (33.3)	0.153
**Pacemaker indication**				
Slow rate atrial fibrillation	158 (40.3)	84 (43.5)	42 (46.9)	0.700
Brady-tachycardia atrial fibrillation	71 (18.1)	42 (21.7)	17 (18.8)	0.639
AV block and permanent atrial fibrillation	49 (12.5)	27 (14.1)	11 (12.5)	0.713
AV block and underlying sinus rhythm	49 (12.5)	10 (5.4)	7 (7.8)	0.426
Sick sinus syndrome	20 (5.1)	17 (8.7)	0	0.002
Other	44 (11.2)	12 (6.5)	13 (14.1)	0.041
**Oral anticoagulation therapy** Vitamin K antagonist Directing-acting anticoagulant **No oral anticoagulation therapy** **Atrial appendage occlusion** Pre-existing Combined strategy	282 (71.9) 90 (22.9) 192 (49.0) 110 (28.1) 12 (7.2) 4 (2.4) 4 (4.8) *	192 / 192 / 2 (1.1)	90 90 / 10 (10.9)	<0.001

* Clinical experience reported by Regoli and colleagues [13].

**Table 2:** Comparison between DOAC anticoagulation regimens.

Complication	Standardized DOAC (Group 1A, *n* = 115)	Other DOAC Regimens (Group 1B, *n* = 77)	*p* Value
**Demographic, clinical**			
**Age, years**	80.5 ± 7.2	81.4 ± 6.6	0.381
**Male**	72 (62.6)	51 (66.2)	0.647
**Structural heart disease**	49 (42.6)	56 (72.7)	<0.001
Ischemic	36 (31.3)	23 (29.9)	
Valvular	10 (8.9)	21 (27.2)	
Other	2 (1.7)	12 (15.6)	
**Hypertension**	98 (85.2)	74 (96.6)	0.016
**Diabetes mellitus**	20 (17.4)	13 (16.9)	1.000
**Renal impairment**	38 (33.0)	51 (66.2)	<0.001
**Dialysis**	0	0	1.000
**COPD**	28 (24.3)	7 (9.1)	0.008
**Peripheral artery disease**	5 (4.3)	16 (20.8)	<0.001
**Previous stroke**	13 (11.3)	11 (14.3)	0.657
**Tumoral disease**	13 (11.3)	13 (16.0)	0.288
**Other comorbidities**	2 (1.7)	2 (2.6)	1.000
**Left ventricular ejection fraction (%)**	55.1 ± 9.3	55.1 ± 9.3	0.819
**Planned hospitalization for implantation**	67 (57.4)	18 (23.4)	<0.001
**Pacemaker indication**			
Slow rate AF	64 (55.7)	25 (32.5)	0.002
Atrial brady-tachi syndrome	28 (24.3)	15 (19.5)	0.482
Atrioventricular block and AF	8 (7.0)	16 (20.8)	0.007
Atrioventricular block and sinus rhythm	2 (1.7)	7 (9.1)	0.031
Sick sinus syndrome	5 (4.3)	10 (13.0)	0.051
Other	8 (7.0)	5 (6.5)	1.000
**Anticoagulation therapy**			
Dabigatran	2 (1.7)	0	0.517
Rivaroxaban	63 (54.7)	21 (27.2)	<0.001
Apixaban	36 (31.3)	39 (50.6)	0.010
Edoxaban	14 (12.2)	17 (22.1)	0.075
**Atrial appendage occlusion**	0	1 (1.2)	1.000

**Table 3:** Procedural and pre-discharge characteristics.

	Standardized DOAC (Gp 1A, *n*= 115)	Other DOAC Regimens (Gp 1B, *n* = 77)	*p* Value
			
**Procedure duration, min**	45.1 ± 14.0	56.2 ± 27.6	<0.001
**Fluoroscopy time, min**	7.0 ± 6.1	9.2 ± 7.5	0.027
			
**Implant success rate**	115 (100)	77 (100)	1.000
			
**DOAC management**			
DOAC stopped (hours)	21.4 ± 5.2	27.0 ± 27.1	0.032
DOAC reinitiation (hours)	14.8 ± 9.3	35.7 ± 33.4	<0.001
			
**Complications**			
**Major**			
Intraprocedural bleeding	3 (2.6)	3 (3.8)	0.685
Pericardial effusion	1	2	
Major femoral access bleeding	2	1	
			
**Minor**			
Puncture site hematoma (<6 cm)	6 (5.2)	3 (3.9)	0.743
**Length of hospital stay, days (IQR)**	3.0 (2.0:3.8)	4.0 (3.0:12.5)	<0.019

## Data Availability

The data presented in this study are available on request from the corresponding author. The data are not publicly available due to restrictions posed by the Cantonal Ethics’ Committee concerning privacy and management of patient data.
